# Prenatal Ultrasonographic Detection of Intracardiac Thrombi in Canine Fetuses With Congenital Heart Defects

**DOI:** 10.1155/crve/1795563

**Published:** 2026-05-30

**Authors:** Federica Valeri, Temy Coppola, Alessandro Troisi, Riccardo Zelli, Lucia Cardinali, Elvio Lepri, Domenico Caivano, Angela Polisca

**Affiliations:** ^1^ Department of Veterinary Medicine, University of Perugia, Perugia, Italy, unipg.it; ^2^ School of Biosciences and Veterinary Medicine, University of Camerino, Matelica, Italy, unicam.it

**Keywords:** canine fetuses, cardiac malformations, thrombosis, ultrasonography

## Abstract

A 5‐year‐old pregnant Newfoundland bitch was evaluated by monitoring fetal viability. Transabdominal ultrasonography of the reproductive tract revealed a homogeneous and hyperechoic mass within the right ventricle associated with abnormal tricuspid valve in a fetus. A similar mass was also visualized in the right atrium of another fetus. The bitch delivered 11 puppies, four of which died after parturition. Necropsy showed tricuspid stenosis in all puppies, with concomitant pulmonary stenosis in one of them. Moreover, an intraventricular thrombus and an interatrial thrombus were each revealed in two puppies. This case series demonstrates the feasibility of prenatal ultrasonographic detection of intracardiac thrombi in canine fetuses and highlights its potential value for early diagnosis of congenital heart defects.

## 1. Introduction

Transabdominal ultrasonography is a well‐established, noninvasive diagnostic technique widely used in small animal reproduction for pregnancy diagnosis, litter size estimation, fetal development assessment, and parturition date prediction [1–4]. In canine patients, this technique can detect pregnancy from 18 to 19 days after the luteinizing hormone (LH) surge, whereas fetal cardiac activity can be identified at 23–24 days post‐LH surge. In veterinary obstetrics, ultrasonography is primarily used to assess fetal heartbeat, and detailed evaluation of cardiac morphology and function is uncommon [5]. In human medicine, prenatal ultrasonography plays a crucial role in the assessment of fetal cardiovascular development and pathology [6, 7].

Congenital heart defects (CHDs) are a major cause of childhood mortality, and prenatal ultrasonography represents the primary diagnostic tool for evaluating fetal cardiovascular anomalies, allowing detection of more than 50% of CHD cases [7]. CHDs are also reported in dogs and may occur either as isolated anomalies or as part of complex syndromes [8]. The most commonly reported CHDs in dogs include pulmonary stenosis (PS), subaortic stenosis, patent ductus arteriosus, and ventricular septal defect, and these are usually diagnosed during the first years of life [8]. Despite this, prenatal identification of CHD in canine fetuses remains rare, largely due to the limited use of advanced fetal echocardiography. Intracardiac thrombus formation in the fetus is an uncommon finding [9] and is rarely reported in veterinary literature [10]. In humans, it is associated with a variety of risk factors, including structural cardiac defects, inherited fetal thrombophilia, hemodynamic disturbances, and maternal or placental disease [11].

We report the ultrasonographic detection of intracardiac thrombi associated with CHD in two Newfoundland fetuses, highlighting the value of detailed fetal cardiac evaluation in dogs. This case series contributes to the limited knowledge of prenatal cardiac pathology in dogs and underscores the potential role of fetal echocardiography as an adjunct diagnostic tool in veterinary obstetrics.

## 2. Case Presentation

A 5‐year‐old pregnant Newfoundland bitch (55 days postovulation) was presented to the Veterinary Teaching Hospital of Perugia University for monitoring of fetal viability. During the ultrasonographic examination (MyLab 30 Gold with a 5.0–8.0‐MHz microconvex transducer, Esaote, Genova, Italy), cardiac abnormalities were detected in two fetuses.

**Figure 1:**
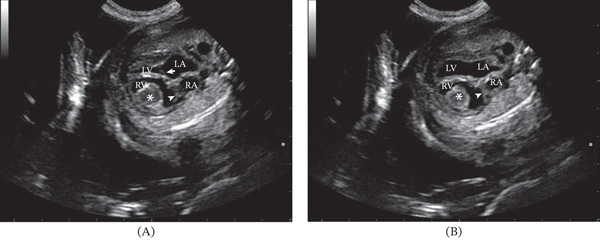
Two‐dimensional long‐axis four‐chamber echocardiographic view of the fetal heart (Case 1). (A) Enlarged right ventricle with a homogeneous and hyperechoic mass inside (∗), thickened tricuspid valve leaflets (arrowhead) compared with mitral valve leaflets (arrow) are evident during the ventricular systole. (B) Fused tricuspid valve leaflets (arrowheads) are evident during the ventricular diastole. LA, left atrium; LV, left ventricle; RA, right atrium; RV, right ventricle.

In one fetus (Case 1), echocardiography revealed a dilated right atrium and ventricle, containing a large, homogeneous, hyperechoic mass within the right ventricular lumen (Figure 1). The tricuspid valve was markedly abnormal, with thickened and fused leaflets (Figure 1). The pulmonary valve could not be clearly visualized. A presumptive diagnosis of right‐sided cardiac valve malformation associated with an intraventricular thrombus was made.

**Figure 2:**
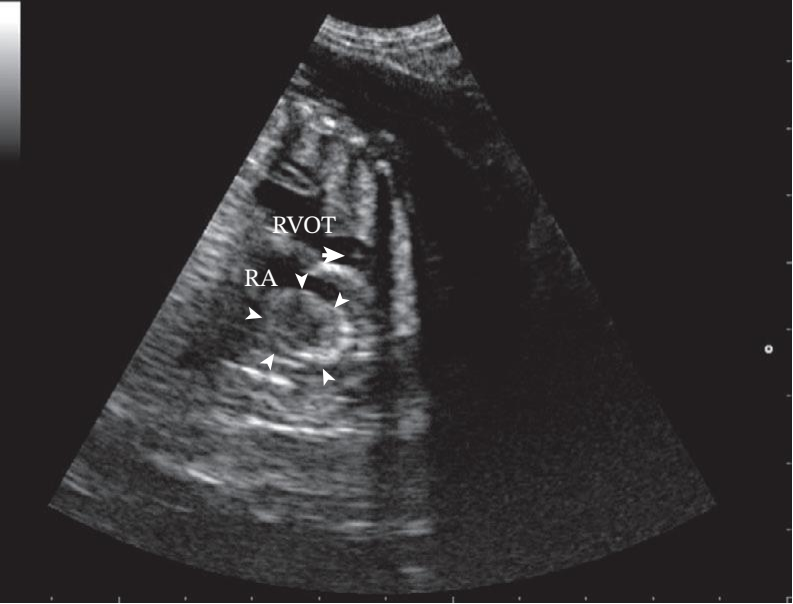
Two‐dimensional short‐axis echocardiographic view of the heart base of the fetus (Case 2). Note the dilated right atrium with a homogeneous and hyperechoic mass inside (arrowheads) and the normal pulmonary valve (arrows). RA, right atrium; RVOT, right ventricle outflow tract.

In the other fetus (Case 2), echocardiography demonstrated a large, homogeneous, hyperechoic mass in the right atrium, associated with severe right atrial enlargement. The pulmonary valve appeared structurally and functionally normal (Figure 2).

Doppler studies were attempted in both affected fetuses; however, the high respiratory rate of the bitch and fetal movements prevented adequate diagnostic imaging. Additionally, the presence of a large litter limited comprehensive echocardiographic evaluation of all fetuses.

The bitch subsequently delivered 11 puppies, four of which died within a few hours after birth.

### 2.1. Necropsy Findings

At necropsy, the four deceased puppies exhibited similar lesions, including diffuse subcutaneous edema, mild serous effusions in the thoracic and abdominal cavities, and an enlarged liver with a mottled surface. The right atrium was markedly enlarged, with a large interatrial communication. The diameter of the right atrioventricular ostium ranged from 1 to 3 mm, with the smallest ostia observed in puppies with intracardiac thrombi; in these cases, the ostium was delineated by a whitish fibrous band to which no valvular leaflets were attached.

**Figure 3:**
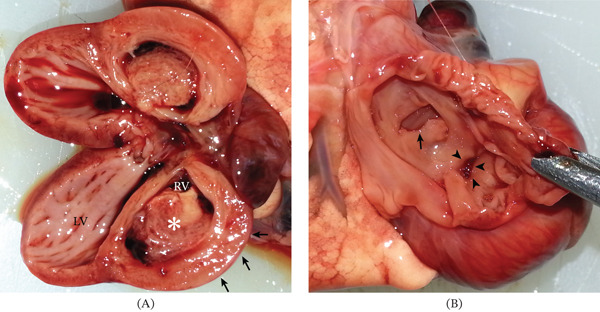
Gross pathology image of the heart in Figure 1. (A) Left and right ventricular view: an intraventricular thrombus (∗) and right ventricle dilation/hypertrophy (arrows) are evident. (B) Right atrial view: dilated right atrium, large atrial septal defect (thin arrow), and stenotic right atrioventricular ostium (arrowheads) are evident. LV, left ventricle; RV, right ventricle.

**Figure 4:**
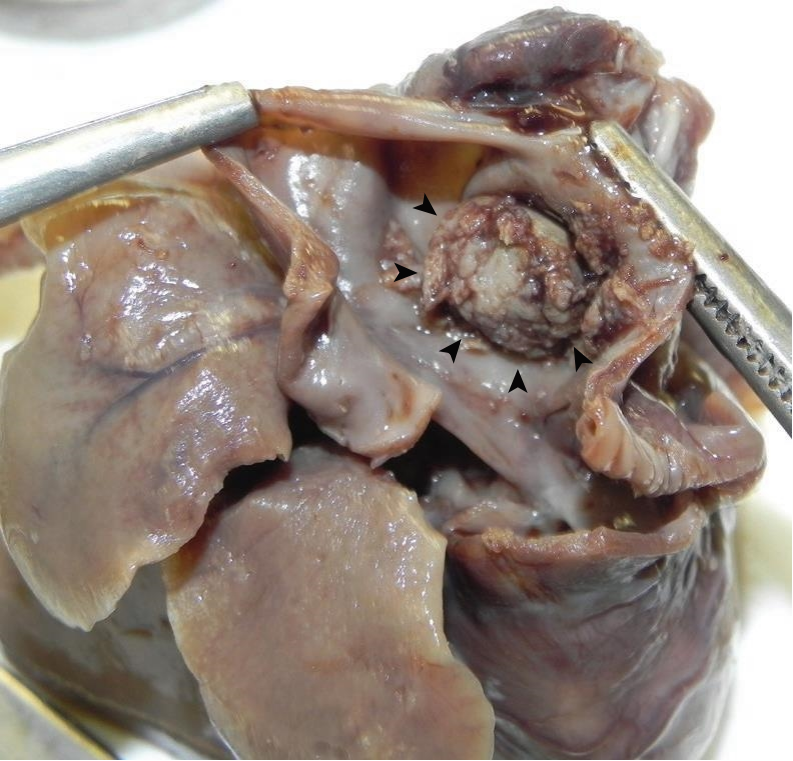
Gross pathology image of the heart in Figure 2. Right atrial view: dilated right atrium with a thrombus inside (arrowheads) is evident.

In one puppy (Case 1), the right ventricle was hypertrophic and dilated, containing a sessile thrombus attached to the ventricular free wall (Figure 3). The pulmonary valve was hypoplastic and stenotic (type B), with poststenotic dilation of the pulmonary trunk. In another puppy (Case 2), the right atrium was dilated and contained a large intracavitary thrombus adherent to the atrial wall, occupying a substantial portion of the chamber (Figure 4).

The pathological diagnosis in all four puppies was tricuspid stenosis (TS) with atrial septal defect (ASD); concomitant type B PS was observed in one case. Furthermore, the necropsy findings confirmed the prenatal ultrasonographic detection of an intraventricular thrombus in Case 1 and an interatrial thrombus in Case 2.

## 3. Discussion

CHDs represent one of the most common causes of morbidity and mortality in dogs under 1 year of age [12]. Tricuspid valve dysplasia and PS have been reported in 3.1% and 32.1% of CHD cases, respectively [8]. ASD is uncommon, with a reported prevalence of 1.1% [8]. Congenital TS due to tricuspid dysplasia is rare, with only a few cases documented in dogs [13–15].

In dogs with TS, restricted leaflet motion and a reduced valve orifice obstruct blood flow from the right atrium to the right ventricle during diastole. This increased resistance results in atrial dilation and right‐sided congestive heart failure [16]. PS is characterized by narrowing of the right ventricular outflow tract to the main pulmonary artery. This obstruction commonly leads to right ventricular hypertrophy, poststenotic dilation of the pulmonary trunk, right atrial enlargement, and right‐sided congestive heart failure [16].

In the present report, all puppies with CHD exhibited TS, and one puppy (Case 1) also had concomitant PS. Additionally, a large ASD in the region of the fossa ovalis was observed in all puppies, likely as a consequence of increased right atrial pressure due to TS. To the authors′ knowledge, very limited information exists regarding the ultrasonographic features of prenatal canine CHD [17].

In this study, CHD was suspected prenatally in two fetuses during ultrasonographic examination. As noted here, a large litter size and frequent fetal movements may complicate comprehensive cardiac evaluation of all fetuses.

Intracardiac thrombosis was observed in Cases 1 and 2 both prenatally via ultrasonography and postmortem. While intracardiac thrombosis is a common complication of acquired cardiac diseases in cats, it is uncommon in dogs. In dogs, thrombus formation in the cardiac chambers has been reported as a complication of atrial fibrillation or myocarditis [18, 19]. Differential diagnoses for the intracardiac masses included thrombosis, tumors, or nonvalvular vegetative endocarditis. Infectious vegetative endocarditis was considered unlikely based on localization (lack of adherence to valvular cusps) and echocardiographic appearance (regular shape, noncauliflower‐like, and without complex echogenicity), leaving thrombosis and neoplasia as the most plausible differentials [20]. Congenital intracardiac tumors are rare in humans and have been rarely reported in dogs [21].

The presence of cardiac valve malformations further increased the likelihood of thrombosis. Dilated cardiac chambers secondary to CHD predispose fetuses to blood stasis and thrombus formation. Additional factors contributing to a prothrombotic state may include endothelial damage and platelet activation due to turbulent blood flow associated with CHD. Notably, both fetuses with intracardiac thrombosis exhibited more severe TS compared with fetuses without thrombi.

To the authors′ knowledge, prenatal ultrasonographic detection of intracardiac masses in canine fetuses has not been previously documented in veterinary literature. The incidence of CHD may be underestimated, as necropsy of stillborn puppies or fetal deaths is not routinely performed. Fetal echocardiographic screening could be a valuable tool to detect forms of CHD that currently go undiagnosed.

Further studies are warranted to evaluate the feasibility, accuracy, and clinical utility of prenatal echocardiography for CHD diagnosis in canine fetuses. In conclusion, prenatal echocardiographic screening may provide a useful method for estimating the true incidence of CHD and could assist in selecting optimal breeding candidates.

## Funding

Open access publishing facilitated by Università degli Studi di Perugia, as part of the Wiley—CRUI‐CARE agreement.

## Conflicts of Interest

The authors declare no conflicts of interest.

## Data Availability

The data that support the findings of this study are available from the corresponding author upon reasonable request. The owner provided informed consent for the inclusion of their animals in this case report and for the processing of related clinical data.
